# The impact of trisomy 21 on epidemiology, management, and outcomes of congenital duodenal obstruction: a population-based study

**DOI:** 10.1007/s00383-020-04628-w

**Published:** 2020-02-29

**Authors:** George S. Bethell, Anna-May Long, Marian Knight, Nigel J. Hall, BAPS-CASS, Abigail Jones, Adil Aslam, Alan Mortell, Amanda McCabe, Andrew Ross, Anna Harris, Anne Lawson, Arun Kelay, Aruna Abhyankar, Ashok Rajimwale, Ashwath Bandi, Atif Saeed, Bala Eradi, Baqer Sharif, Brian MacCormack, Caroline Pardy, Catherine Ridd, Ceri Jones, Ceri Jones, Cezar Nicoara, Chris Driver, Chris Parsons, Chun-Sui Kwok, Clare Rees, Clare Skerritt, Damian Magnie, Dan Aronson, David Marshall, Dawn Deacy, Debasish Banerjee, Diane De Caluwe, Dorothy Kufeji, Eleri Cusick, Elizabeth O’Connor, Georgina Bough, Gergana Racheva, Govind Murthi, Harmit Ghattaura, Hetal Patel, Ian Jones, Ian Sugarman, Ike Njere, Ingo Jester, Jonathan Durell, Joseph Davidson, Keren Sloan, Kevin Cao, Khalid Elmalik, Lee Smith, Leel Nellihela, Lopes, Lucinda Tullie, Lucy Henderson, Madhavi Kakade, Maryam Haneef, Melania Matcovici, Michael Dawrant, Michelle Horridue, Miguel Soares-Oliveira, Miriam Doyle, Mohamed Shalaby, Morven Allan, Oliver Burdell, Paul Charlesworth, Paul Johnson, Rahman Halim, Richard Hill, Riyad Peeraully, Rosie Cresner, Ross Craigie, Samir Gupta, Sandeep Motiwale, Sanja Besarovic, Saravanakumar Paramalingam, Sean Marven, Shailesh Patel, Shazia Sharif, Shehryer Naqvi, Simon Clarke, Simon Kenny, Sofia Eriksson, Susan Payne, Thanos Tyraskis, Thomas Tsang, Tim Bradnock, William Calvert, Yatin Patel

**Affiliations:** 1 University Surgery Unit, Faculty of Medicine, University of Southampton, Southampton, SO16 6YD UK; 2 National Perinatal Epidemiology Unit, Oxford, UK; 3 Department of Paediatric Surgery, Cambridge University Hospitals, Cambridge, UK

**Keywords:** Duodenal atresia, Duodenal stenosis, Down syndrome, Trisomy 21, Congenital cardiac disease

## Abstract

**Purpose:**

Congenital duodenal obstruction (CDO) is associated with trisomy 21 (T21), or Down’s syndrome, in around a third of infants. The aim of this study was to explore the impact of T21 on the epidemiology, management, and outcomes of infants with CDO.

**Methods:**

Data were prospectively collected from specialist neonatal surgical centres in the United Kingdom over a 12 month period from March 2016 using established population-based methodology for all babies with CDO. Infants with T21 were compared to those without any chromosomal anomaly.

**Results:**

Of 102 infants with CDO that underwent operative repair, T21 was present in 33 [32% (95% CI 23–41%)] babies. Cardiac anomalies were more common in those with T21 compared to those without a chromosomal anomaly (91 vs 17%, *p* < 0.001), whereas associated gastrointestinal anomalies were less common in infants with T21 (3 vs 12%, *p* = 0.03). Surgical management was not influenced by T21. Time to achieve full enteral feed, need for repeat related surgery, and mortality were similar between groups. Infants with T21 had a longer median initial inpatient stay (23 vs 16.5 days, *p* = 0.02).

**Conclusions:**

Infants with T21 have a higher incidence of cardiac anomalies and a longer initial inpatient stay; however, it does not change CDO management or outcomes. This information is important for prenatal and postnatal counselling of parents of infants with CDO and T21.

## Introduction

Congenital duodenal obstruction (CDO), due to duodenal atresia or stenosis, is seen in 1.22 per 10 000 live births and requires surgical restoration of gastrointestinal tract continuity which usually takes place within the first few days of life [1]. Trisomy 21 (T21), or Down’s syndrome, is present in around a third of infants with CDO and is, therefore, the most commonly associated chromosomal anomaly [1, 2].

In some other neonatal surgical conditions, infants with T21 are managed differently than those without T21 possibly due to concerns about tissue healing. For example, in a recent observational study of the management of infants with Hirschsprung disease in the United Kingdom (UK), infants with T21 were four times more likely to undergo enterostomy formation prior to definitive surgical management than those without a chromosomal anomaly [3].

Previous, single-centre studies that have explored the impact of T21 on the management of CDO have produced conflicting data. A study from Thailand found that infants with T21 had a higher rate of post-operative complications and mortality [4]. However, a UK-based institution reported no difference in outcomes of CDO between those with and without T21 [2].

Given the conflicting, low-quality, existing evidence on this topic the aim of this study was to explore the impact of T21 on the epidemiology, management, and outcomes of infants with CDO.

## Methods

This analysis was undertaken according to a pre-specified protocol using the British Association of Paediatric Surgeons Congenital Anomaly Surveillance System. Ethical approval was granted by the National Research Ethics Service South Central-Oxford A committee (ref: 12/SC/0416).

### Case identification

The process of case identification has been described previously [1]. Briefly, live born cases of congenital occlusion or narrowing of the duodenum associated with atresia, stenosis, duodenal web, or annular pancreas presenting prior to a post-conceptual age of 44 completed weeks were prospectively identified over a 1-year period from 1st March 2016 at all 28 specialist neonatal surgical centres in the UK. Cases of duodenal occlusion or narrowing caused by congenital bands associated with malrotation, intestinal volvulus, duplication cyst, or malignancy without an intrinsic duodenal abnormality were excluded.

### Data collection

After a case was identified via a monthly reporting card, a data collection form was sent for each case to the specialist neonatal surgical centre at day 28 and then 1 year following surgical repair. These forms were then returned and data were then entered into a database at the National Perinatal Epidemiological Unit, Oxford.

For the purpose of this analysis, infants were only included if they underwent operative repair, had a confirmed diagnosis of T21, or had no other detected chromosomal abnormality.

### Outcomes

Main outcomes were defined in the study protocol and were time to achieve full enteral feeds, use and duration of parenteral nutrition, number of central venous catheters (CVCs—including both peripherally inserted and centrally inserted catheters) used, CVC-related complications, anastomotic complications, inpatient hospital stay, standardised weight gain/loss, and death.

### Statistical analysis

Statistical analysis took place using StataSE v15 (StataCorp LLC, Texas, USA). Fisher’s exact test was used for categorical data and Chi-squared test was used for categorical data with more than 2 × 2 analysis. A Mann–Whitney *U* test was used for continuous data. Data are reported as median with range or number with percentage as appropriate. *p* < 0.05 was considered statistically significant.

To calculate standardised weight change, the *zanthro* package for StataSE v15 was used to calculate weight-for-age *z* scores using UK World Health Organization term and preterm growth reference charts. For infants with Down syndrome, the *Zemel 2015* weight-for-age growth chart was used instead [5]. The weight-for-age *z* score, also known as standard deviation (SD) score, is a measure of the SD of weight from the mean value of a reference population matched for gestational age and sex [6].

## Results

### Study population

In the study period, there were 103 babies with CDO and 102 that underwent operative repair. T21 was present in 33 [32% (95% CI 23–41%)] infants. In 65 (63%) infants with CDO, there was no chromosomal anomaly reported and death occurred prior to operative repair once. Additionally, there were five (4.9%) babies with chromosomal anomalies other than T21; these were excluded along with the one infant who died prior to operative repair. The study population, therefore, comprised 33 infants with T21 and 64 infants without a chromosomal anomaly. Of those alive at 28 days following surgical repair (95/97 infants), follow-up data at 1 year were available for 76 (80%) infants including 25/33 (81%) with T21 and 51/64 (80%) with no chromosomal anomaly.

### Prenatal screening and demographics

Prenatal screening for chromosomal anomalies was undertaken in 23 (24%) cases using either amniocentesis (*n* = 17), non-invasive prenatal testing of maternal blood (*n* = 4), chorionic villi sampling (*n* = 1), or microarray comparative genomic hybridisation (*n* = 1). Out of those tested T21 was detected in 11 (48%) foetuses. Therefore, the overall prenatal detection rate of T21 in CDO was 33% (95% CI 17–49%). The sex, gestational age at birth, birthweight, atresia type, and site of obstruction were all similar between those with T21 and those without chromosomal anomaly (Table 1).

**Table 1:** Group characteristics and management undertaken comparing infants with T21 to those without a chromosomal anomaly

	T21 (*n* = 33)	No chromosomal anomaly (*n* = 64)	*p*
Male, *n* (%)	18 (55)	35 (55)	1.00
Gestational age at birth, weeks (range)	36.3 (28.1–39.4)	36.3 (25.6–42.3)	0.61
Birthweight, grams (range)	2290 (800–3730)	2520 (830–4320)	0.38
Prenatal CDO diagnosis, n (%)	24 (73)	32 (50)	**0.05**
Atresia type, *n* (%)			
I	15 (45)	24 (38)	0.43
II	3 (9.1)	2 (3.1)	
III	9 (27.3)	25 (39)	
Not reported or not identified	6 (18.2)	13 (20)	
Site of obstruction, n (%)			
Pre-ampullary	6 (18)	19 (30)	0.13
Post-ampullary	13 (39)	30 (47)	
Not reported or not identified	14 (42)	15 (23)	
Age at surgery, days (range)	2 (0–14)	3 (0–75)	0.08
Repair type, *n* (%)			
Duodenoduodenostomy	24 (75)	49 (77)	0.15
Duodenojejunostomy	8 (25)	7 (11)	
Membrane incision	0 (0)	1 (1.6)	
Membrane resection	0 (0)	4 (6.3)	
Duodenoplasty	0 (0)	3 (4.7)	
TAT used, *n* (%)	16 (49)	24 (38)	0.38
PICC/CVC used, *n* (%)	29 (88)	59 (92)	0.48
PN used, *n* (%)	28 (85)	58 (91)	0.50

*p* value in bold indicates statistically significant

*T21* trisomy 21, *CDO* congenital duodenal obstruction, *TAT* trans-anastomotic tube, *PICC* peripherally inserted central catheter, *CVC* central venous catheter, *PN* parenteral nutrition

### Associated anomalies

Other congenital anomalies were present in 93% (31/33) of infants with T21 and 50% (32/64) infants without chromosomal anomaly. Three of these anomalies were not reported at 28 days, but were reported at 1 year (two with atrial septal defects and one with annular pancreas). Cardiac anomalies were the most frequent and were seen more often in those with T21 than those without chromosomal anomaly (91 vs 27%, *p* < 0.001). Other gastrointestinal (GI) tract anomalies were more common in those without chromosomal anomaly compared to those with T21 (Table 2). There were no cases of Hirschsprung disease identified in either group.

**Table 2:** Associated anomalies with CDO comparing infants with T21 to those without a chromosomal anomaly

Associated anomalies (*n* = 63^a^)	T21 (*n* = 33)	No chromosomal anomaly (*n* = 64)	*p*
Associated cardiac anomaly	30 (91)	17 (27)	**< 0.001**
Isolated PDA	3 (9.1)	2 (3)	
PDA with other structural cardiac anomaly	14 (42)	6 (9)	
VSD	12 (36)	5 (7.8)	
PFO	8 (24)	6 (9.4)	
ASD	8 (24)	4 (6.3)	
AVSD	4 (12)	0 (0)	
Tetralogy of fallot	2 (6.1)	1 (1.6)	
Coarctation/hypoplasia of aorta	1 (3.0)	1 (1.6)	
Other	3 (9.1)	7 (11)	
Annular pancreas	1 (3.0)	11 (17.2)	0.05^$^
Biliary tree anomaly	0 (0)	0 (0)	
Abnormal midgut rotation	7 (21)	15 (23)	1.00
Other gastrointestinal anomaly	1 (3.0)	12 (20)	**0.03**
Anorectal malformation	1 (3.0)	4 (6.3)	
EA with TEF	0 (0)	5 (7.8)	
Isolated EA	0 (0)	4 (6.3)	
Meckel’s diverticulum	0 (0)	1 (1.6)	
Ileal atresia	0 (0)	2 (3.1)	
Cloaca anomaly	0 (0)	1 (1.6)	
Other structural anomalies	4 (12)	12 (19)	0.41
Renal	0 (0)	5 (7.8)	
Limb	1 (3.0)	1 (1.6)	
Spine	0 (0)	2 (3.1)	
Other	3 (9.1)	7 (11)	

*p* values in bold indicate statistically significant

*T21* trisomy 21, *PDA* patent ductus arteriosus, *VSD* ventricular septal defect, *PFO* patent foramen ovale, *ASD* atrial septal defect, *AVSD* atrioventricular septal defect, *EA* esophageal atresia, and *TEF* tracheoesophageal fistula

^a^Note infants may have multiple anomalies; therefore, figures add up to more than 100%

^$^denotes value which rounds to 0.05 and, therefore, not statistically significant

### Anatomy and management of CDO

The type of duodenal atresia or stenosis along with the site of obstruction was similar between those with T21 and without chromosomal anomaly (Table 1). Age at surgical repair, surgical technique used, approach to post-operative feeding, and nutritional management were all similar between the groups; however, those with T21 had a shorter time to commencing enteral feeds post-repair than those without (2.5 vs 4 days, *p* = 0.046).

### Outcome

The proportion of infants who had achieved full enteral feeds at both 28 days and 1 year following surgical repair was similar between those with T21 and those with no chromosomal anomaly (Table 3). Overall duration of parenteral nutrition (PN) and number of infants experiencing CVC complications were similar between the two groups.

**Table 3:** Outcomes comparing infants with T21 to those without a chromosomal anomaly at either 28 days or 1-year post surgical repair of CDO

	T21 (*n* = 33)	No chromosomal anomaly (*n* = 64)	*p*
Mortality at 28 days, *n* (%)	2 (6.1)	0 (0)	0.11
Achieved full enteral feeds at 28 days^a^, *n* (%)	27 (93)	53 (87)	0.49
PN at 28 days post op^a^, *n* (%)	2 (6.5)	7 (11)	0.71
Discharged home at 28 days^a^, *n* (%)	16 (67)	40 (78)	0.39
Standardised weight change—birth to 28 days, z score (range)	− 0.37 (− 1.15-1.11)	− 0.80 (− 2.34–0.53)	**0.001**
Mortality at 1 year^c^, *n* (%)	4 (15)	2 (4)	0.18
Achieved full enteral feeds at 1 year^b^, *n* (%)	20 (100)	43 (98)	1.00
Time to full enteral feeds post op, days (range)	12.5 (4–37)	13 (5–44)	0.33
PN at 1 year post op^b^, *n* (%)	0 (0)	1 (2.0)	1.00
PN duration, days (range)	12 (2–35)	11 (2–134)	0.94
Discharged home at 1 year^b^, *n* (%)	21 (100)	45 (100)	1.00
Inpatient stay post op, days (range)	23 (11-114)	16.5 (6–149)	**0.02**
Repeat surgery related to CDO^c^, *n* (%)	1 (3.7)	4 (7.8)	0.65
PICC/CVC-related complication^c^, *n* (%)	7 (25)	14 (26)	1.00
Standardised weight change—birth to 1 year, *z* score (range)	0.68 (− 2.12 to 2.56)	− 0.33 (− 2.57 to 2.23)	**0.01**

*p* values in bold indicate statistically significant

*T21* trisomy 21, *PN* parenteral nutrition, *PICC* peripherally inserted central catheter, *CVC* central venous catheter, *CDO* congenital duodenal obstruction

^a^Excluded if infant died before 28-days post surgical repair or missing data

^b^Excluded if infant died before 1-year post surgical repair, missing data or missing 1-year follow-up

^c^Excluded if event status unknown at 1-year follow-up

In total, there were ten post-operative complications, and these were small bowel obstruction (*n* = 3), wound infection (*n* = 2), anastomotic leak (*n* = 1), chest sepsis requiring intubation (*n* = 1), wound dehiscence (*n* = 1), incisional hernia (*n* = 1), and a stitch abscess (*n* = 1). These complications were distributed evenly between those with T21 and infants without a chromosomal anomaly (11% vs 13%*, p* = 1.00). In total, there were five repeat laparotomies which were similarly distributed between the two groups of infants (Table 3).

Those with T21 had a longer inpatient stay than those without chromosomal anomaly (23 vs 16.5 days, *p* = 0.02), but all infants alive with follow-up at 1 year following surgical repair had been discharged by this time.

Difference in standardised weight-for-age *z* scores from birth was calculated for the two groups at both 28 days and 1 year post surgical repair and those with T21 has higher *z* scores than those without chromosomal anomaly at both time points (Table 3). There were 6 (7.8%) deaths within 1 year from causes unrelated to CDO. Although the mortality rate was higher in infants with T21 (15 vs 4%), this did not reach statistical significance, noting the limited statistical power of this comparison. Two of these deaths occurred within 28 days of operative repair.

## Discussion

We and others have identified that T21 is present in around one-third of infants with CDO [1, 7]. T21 is, therefore, the most commonly associated chromosomal anomaly. Since it is known that in other conditions managed by paediatric surgeons, management differs for those with T21 compared to those without a chromosomal anomaly such as T21 [3], we aimed to explore the impact of T21 on the epidemiology, management, and outcomes of those with CDO. Our key finding from this prospective population-based study is that we found very little difference in epidemiology and management and outcomes of these infants with the exceptions that infants with T21 were more likely to have CDO diagnosed antenatally, more likely to have a cardiac anomaly, and less likely to have a GI tract anomaly than infants without chromosomal abnormality. Furthermore, infants with T21 had a longer initial length of hospital stay.

Non-invasive prenatal testing (NIPT) has been introduced as a screening tool for various chromosomal anomalies including T21 which can be undertaken early in fetal life without risk to the pregnancy which is associated with the traditional methods such as amniocentesis [8]. Meta-analysis has shown that in the general obstetric population, NIPT can achieve a sensitivity of 95.9% and a specificity of 99.9% for detecting T21 [9]. Despite this relatively new technology, the majority of foetuses tested for chromosomal anomalies in this study did so via amniocentesis. Only a third of infants with T21 and CDO was the T21 diagnosis detected prenatally. This figure is lower than we might anticipate given the recognised association between T21 and CDO. It is possible that some foetuses with T21 detected prenatally were terminated in utero (and, therefore, were never included in this study) or that families elected not to screen for T21 despite the presence of features suggestive of CDO. Conversely infants with T21 were more likely to have a diagnosis of CDO made antenatally than those without T21. We speculate that it is likely that infants with T21 had additional third trimester ultrasound scans, thereby providing an additional opportunity for CDO to be detected.

Cardiac anomalies are the most common anomaly associated with CDO, and in this study, they were seen in over 90% of infants with T21 which is slightly more than the 81.5% reported by Singh et al. in a similar study [1, 2]. Cardiac anomalies in T21 are reported to be present in 33–56% of babies, and therefore, these are more prevalent in T21 with CDO [10]. It is not clear from the data collected whether these cardiac anomalies were detected antenatally on fetal ultrasonography or whether these were diagnosed in the postnatal period. Additionally, there were two atrial septal defects (ASD) not identified by day 28 in this study and, therefore, reported at 1 year following repair of CDO. These findings highlight the importance of careful screening for cardiac anomalies with fetal ultrasonography in suspected babies with CDO. We would also recommend early echocardiography following birth in these infants, since the risk of congenital cardiac anomaly is particularly high.

After congenital cardiac anomalies, GI tract anomalies are the second most commonly associated anomaly in T21, and of these, CDO is the most common type of GI tract anomaly. Previous data report that 2.6–14.6% of live births with T21 will have CDO [10–12]. Associated GI tract anomalies, excluding abnormal gut rotation, were rare in those with T21 which is similar to findings from a previous study [2]. Despite a recognised association between T21 and Hirschsprung disease, no infant in this study had both CDO and Hirschsprung disease.

Two previous single-centre retrospective studies have reported outcomes of infants with CDO and T21. One [4] was from a centre in Thailand including 227 patients over a 10-year period ending in 2006 and another [2] was from the UK including 79 infants over an 11-year period ending in 2002. In this current study, there was no difference detected in outcomes between those with T21 and those with no chromosomal anomaly except that those with T21 have a longer inpatient stay and that those with T21 have better standardised weight gain. Singh et al. found no differences in terms of enteral feeding, post-operative complications, or mortality between the two groups. They also reported a similar reoperation rate for reasons related to CDO (7.8% vs 5.2% in this series). Niramis et al. in a different healthcare setting and era found that those with T21 were more likely to undergo duodenoduodenostomy than an alternate procedure and mortality was higher in the T21 group. Complications were also more frequent in the T21 group, but no information was provided on feeding outcomes.

In this study, the difference in standardised weight-for-age *z* scores has been calculated from birth to 28 days and 1-year post surgical repair. At 28 days, standardised weight loss was greater in the group without chromosomal anomaly, and then, at 1 year, those with T21 were thriving compared to those without chromosomal anomaly. There are multiple possible explanations for this: first, there were significantly more associated other GI tract anomalies in the group without T21 which may have influenced feeding; second, growth charts for those with T21 expect less weight gain than those without chromosomal anomaly. Additionally, it is possible that the prolonged hospital stay experienced by those with T21 resulted in increased nutritional attention. Regardless of the explanation for this finding, it is reassuring for parents and clinicians of those with T21.

A strength of this study is the use of proven surveillance methodology for case ascertainment and a high 1-year follow-up rate (80%) [1]. Data were collected prospectively from multiple neonatal surgical centres over a short period of time and, therefore, represent contemporary practice. This work is limited in that we report relatively short follow-up (1 year), and given its observational nature, it is possible that some outcomes were influenced by associated anomalies other than T21.

## Conclusion

This national population-based study of infants with CDO demonstrates that infants with T21 are managed similarly to their counterparts without T21 and have similar, and good, short-term outcomes. Infants with T21 have a higher incidence of cardiac anomaly and a longer length of initial hospital stay, factors that may be related. These data can be used by clinicians for both prenatal and postnatal counselling.
